# Hybrid Surgery Options for Complex Clinical Scenarios in Adult Patients with Congenital Heart Disease: Three Case Reports

**DOI:** 10.3389/fsurg.2017.00007

**Published:** 2017-02-09

**Authors:** Filippo Rapetto, Damien Kenny, Mark Turner, Andrew Parry, Serban Stoica, Orhan Uzun, Massimo Caputo

**Affiliations:** ^1^Department of Cardiac Surgery, Bristol Heart Institute, University of Bristol, Bristol, UK; ^2^Department of Cardiac Surgery, Bristol Royal Hospital for Children, University of Bristol, Bristol, UK; ^3^Department of Paediatric Cardiology, Rush University Medical Center, Chicago, IL, USA; ^4^Department of Cardiology, Bristol Heart Institute, University of Bristol, Bristol, UK; ^5^Department of Paediatric Cardiology, University Hospital of Wales, Cardiff, UK

**Keywords:** ACHD, cardiac surgery, hybrid surgery, pulmonary valve replacement, paravalvular leak

## Abstract

The strategy for the management of adult patients with congenital heart disease (CHD) often represents a challenge for cardiac surgeons and cardiologists due to complex anatomy, wide range of clinical presentations, and a high-risk profile. However, hybrid approach may represent an attractive solution. We report three cases of adult patients previously operated for CHD and recently treated with a hybrid approach in our institution. Case 1: a 76-year-old woman with permanent atrial fibrillation, lung disease, chronic kidney disease, microcytic anemia, and type II diabetes mellitus, previously operated for atrial septal defect closure and pulmonary valvotomy, presented with severe pulmonary regurgitation and advanced right ventricular failure. In order to minimize the surgical risk, a hybrid approach was used: an extensive right ventricular outflow tract (RVOT) plication was followed by implantation of an Edwards Sapien XT prosthesis in the RVOT through the right ventricular apex, without cardiopulmonary bypass. Case 2: a 64-year-old man with previous atrial septum excision and pericardial baffle for partial anomalous pulmonary venous drainage with intact interatrial septum, presented with worsening dyspnea, right ventricular failure, and pulmonary hypertension caused by baffle stenosis. His comorbidities included coronary artery disease, atrial flutter, and previous left pneumonectomy. After performing a redo longitudinal median sternotomy, a 20-mm stent was implanted in the baffle with access through the superior vena cava. Case 3: a 50-year-old man, with previous atrioventricular septal defect repair, followed by mitral valve replacement with a mechanical prosthesis, subsequently developed a paravalvular leak (PVL) with severe mitral regurgitation and severe left ventricular dysfunction. He underwent a transapical PVL device closure with two Amplatzer Vascular Plugs. In our opinion, hybrid surgery is a promising therapeutic modality that increases the available treatment options for this patient population. A multidisciplinary and patient-tailored approach is crucial in these complex clinical scenarios.

## Introduction

The surgical and cardiological approach to patients with congenital heart disease (CHD) has been constantly evolving, and the life expectancy of this cohort of patients is increasing. Therefore, a significant number of adult patients with CHD, complex anatomy, and high-risk profile need multiple operations throughout their life. In this context, reoperations often carry a considerable morbidity and mortality risk that is determined both by technical issues and by the potentially detrimental effects of cardiopulmonary bypass (CPB) and cardioplegic arrest.

Hybrid procedures performed in dedicated theaters by a team of cardiac surgeons and cardiologists have been developed in order to minimize the surgical risk in specific patient populations (1), and they are currently widely adopted in several fields of both adult and pediatric cardiac surgery such as aortic arch disease, hypoplastic left heart syndrome, and ventricular septal defect closure (2, 3).

We have been increasingly performing hybrid procedures also in adult patients with CHD at Bristol Heart Institute. Here, we report three cases of patients who previously underwent cardiac operations for CHD and who have been successfully treated with a hybrid approach.

## Case Reports

### Case 1

A 76-year-old woman underwent a secundum atrial septal defect (ASD) closure and pulmonary valvotomy at the age of 32 at another institution. She presented with severe pulmonary regurgitation associated with clinical and echocardiographic signs of advanced right ventricular failure, including diffuse peripheral edema and a dilated right ventricle with septal flattening and moderate tricuspid regurgitation; the main pulmonary artery diameter was 47 mm. Her chest X-ray showed an enlarged heart and main pulmonary arteries. Cardiac catheterization and cardiac magnetic resonance imaging demonstrated a dilated main pulmonary artery and branch pulmonary arteries.

This patient had a past medical history of diabetes mellitus, peripheral vascular disease associated with stage 3 chronic kidney disease (CKD), and bowel angiodysplasia with symptomatic microcytic anemia. She also had undergone an unsuccessful catheter ablation for non-isthmus-dependent atrial flutter, and she was chronically anticoagulated with warfarin when presented to our team.

The estimated glomerular filtration rate on preoperative blood tests was 39 ml/min/1.73 m^2^.

In order to minimize the surgical risk and to avoid the potential complications of CPB in a patient with chronic bleeding, peripheral vascular disease, and CKD, it was decided to hybridly implant an Edwards Sapien XT prosthesis (Edwards Lifesciences, Irvine, CA, USA) in the right ventricular outflow tract (RVOT) within the native pulmonary valve.

**Figure 1:**
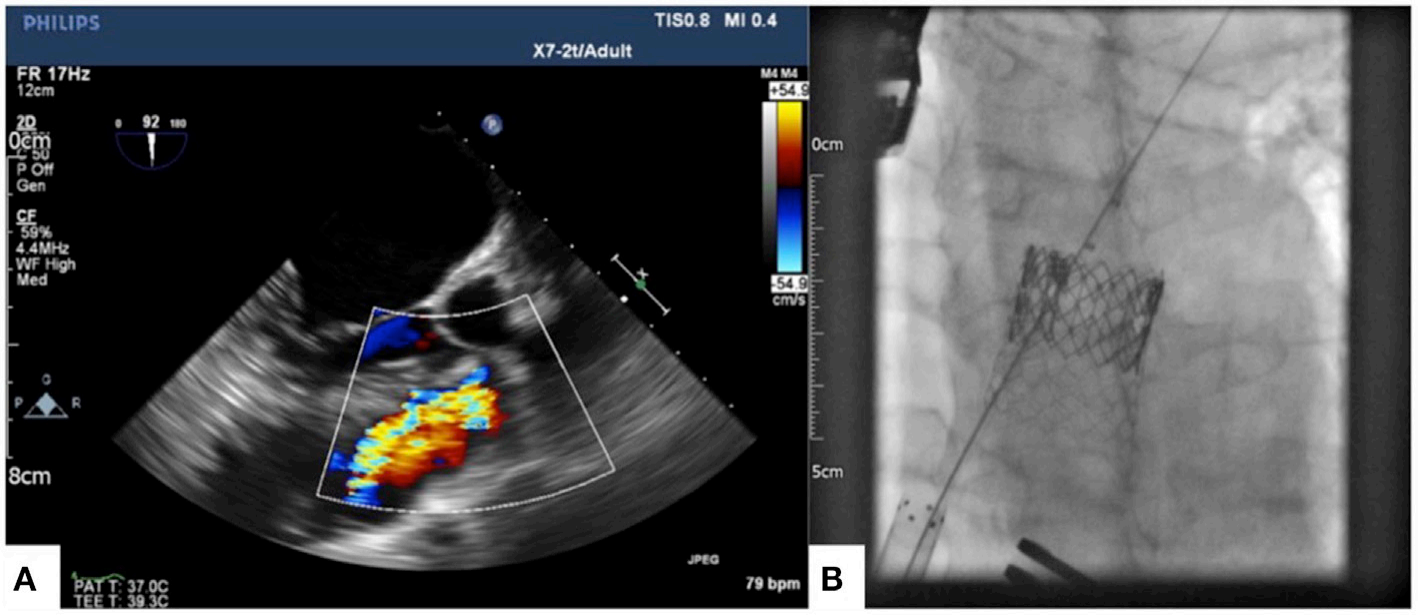
**Baseline pulmonary regurgitation showed by intraoperative transesophageal echocardiography (A) and 29-mm Edwards Sapien XT prosthesis within an Andramed AS43XXL stent implanted in the pulmonary position (B)**.

A redo longitudinal median sternotomy and an extensive longitudinal plication of the main pulmonary artery and infundibular area were performed using a continuous 3-0 Prolene stitch reinforced with two Teflon strips. Transesophageal and epicardial echocardiogram, as well as angiography, were used to confirm that the RVOT had been adequately downsized prior to proceeding with transcatheter pulmonary valve implantation (TPVI). A purse string suture was then placed on the anterior surface of the right ventricle. Through this perventricular access, an Andramed AndraStent AS43XXL stent (Andramed GmbH, Reutlingen, Germany) premounted on a 28 mm × 5 cm BIB balloon (PFM Medical, Koln, Germany) was implanted in the RVOT; the inner balloon was inflated to 4 Atm, the outer balloon was inflated to 2 Atm. Finally, a 29-mm Edwards Sapien XT prosthesis was positioned within the stent (Figures 1A,B show baseline pulmonary regurgitation on transesophageal echocardiography and final result on fluoroscopy, respectively).

A routine transthoracic echocardiogram was performed 5 days after the procedure, and it demonstrated moderate right ventricular impairment and low gradient across the pulmonary prosthesis (maximum velocity 1.8 m/s, mean gradient 7 mmHg, maximum gradient 13 mmHg) with no pulmonary regurgitation. The patient was discharged home 13 days postoperatively.

### Case 2

A 64-year-old man underwent repair of partial right-sided anomalous pulmonary venous drainage (PAPVD) with intact interatrial septum. At this operation, the atrial septum was excised to create a pericardial baffle for the pulmonary veins to drain into the left atrium. The anomalous pulmonary veins entered the superior vena cava high above the level of the right pulmonary artery; therefore, the superior vena cava transection and relocation approach had been chosen to correct the defect. Over time, the atrial septum had grown back, therefore causing pulmonary venous stenosis. As a consequence, the patient presented with NYHA class III dyspnea, right ventricular failure, and pulmonary hypertension.

**Figure 2:**
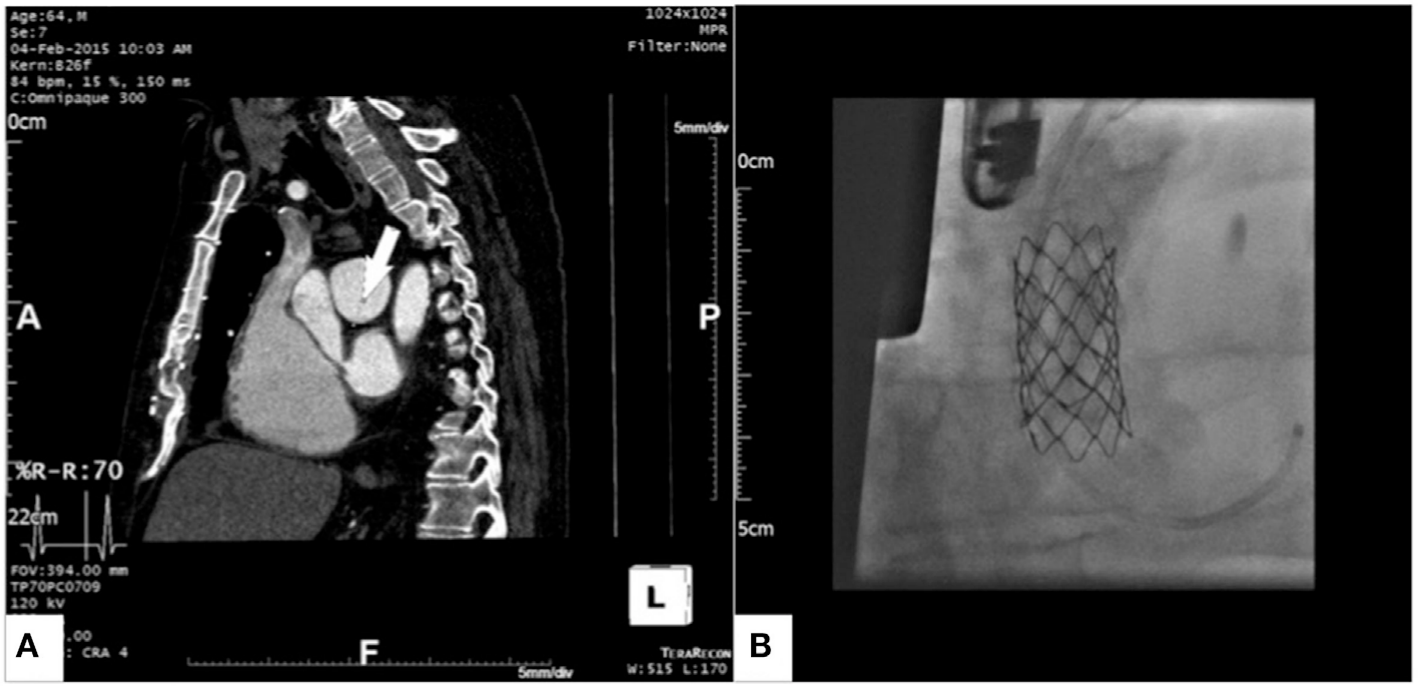
**Gated computed tomography scan showing severe stenosis at the left atrial end of the pericardial baffle [(A), arrow] and 16 mm × 45 mm Premounted CP Stent expanded within the pericardial baffle [(B), the sheath can be seen in the stump of the superior vena cava]**.

The preoperative echocardiogram showed a severely dilated right heart with a tricuspid annulus of 58 mm, severe pulmonary hypertension suggested by a tricuspid regurgitation jet velocity of 4 m/s, and normal left ventricular function. A preoperative computed tomography scan was performed, and it demonstrated a severely dilated right ventricle (end-diastolic diameter of 68 mm) and a severe stenosis at the distal end of the baffle (Figure 2A). Mild coronary artery disease was also present, and it was confirmed by the preoperative coronary angiogram.

The patient had several comorbidities, including type II diabetes mellitus and paroxysmal atrial fibrillation on Warfarin; furthermore, he had undergone a left pneumonectomy at the age of 4 due to rubella, and he had subsequently experienced recurrent low respiratory tract infections on his right lung.

Having considered the comorbidities and the high risk for complications on the right lung that a conventional open heart reoperation would have carried, it was decided to address the pericardial baffle stenosis through an interventional hybrid approach. In order to gain access to the pulmonary venous system, a redo longitudinal median sternotomy was performed, and after dissection of the adhesions a purse string suture was placed on the stump of the native superior vena cava. A 14 F sheath was then inserted and a 16 mm × 45 mm Premounted CP Stent (PFM Medical, Koln, Germany) was implanted within the pericardial baffle on the beating heart and avoiding CPB. The stent was then post-dilated with a 20-mm Cristal balloon (BALT Extrusion, Montmorency, France). Transesophageal echo and angiography were used to confirm the good result; no gradient across the stent was measured. The final result is shown in Figure 2B.

The early postoperative course was uneventful. The patient was electively readmitted to our institution 6 months after the procedure to have a cardiac catheterization as part of the workup for his pulmonary hypertension. This demonstrated pulmonary hypertension secondary to lung disease and confirmed the baffle stent to be patent, showing half-systemic pulmonary artery pressure with a calculated pulmonary vascular resistance of 4 Wood Units, a pulmonary capillary wedge pressure of 13 mmHg, and a mean gradient across the stent (between pulmonary capillary wedge pressure and left ventricular end-diastolic pressure) of 5 mmHg.

### Case 3

A 50-year-old man previously undergoing primum ASD patch closure and left atrioventricular valve cleft repair at the age of 6, developed left atrioventricular valve regurgitation, and the valve was replaced with a 29-mm mechanical prosthesis at the age of 20.

His past medical history also included permanent atrial fibrillation with complete heart block and need for permanent pacemaker at the age of 40. The patient then developed severe left ventricular dysfunction, and his pacemaker was upgraded to a biventricular defibrillator (CRT-D) at the age of 46.

He presented to our team with worsening dyspnea, cardiac failure, and anemia. A transthoracic echocardiogram showed severe left atrioventricular valve paravalvular regurgitation, moderate/severe left ventricular dysfunction, severe tricuspid regurgitation, and moderate pulmonary hypertension; the mean gradient across the mechanical prosthesis was 6 mmHg. A left and right cardiac catheterization with coronary angiogram confirmed pulmonary hypertension and demonstrated normal coronary arteries.

The preoperative blood tests suggested hemolysis to be the cause of the anemia, with increased lactate dehydrogenase (LDH) and reduced haptoglobin; hemoglobinuria was also found.

The transthoracic echo was assessed by our team, and it was decided to address the paravalvular leak (PVL) with a transapical trancatheter approach; this approach allowed avoidance of a third sternotomy, CPB, and cardioplegic cardiac arrest in a patient with significantly depressed left ventricular ejection fraction.

**Figure 3:**
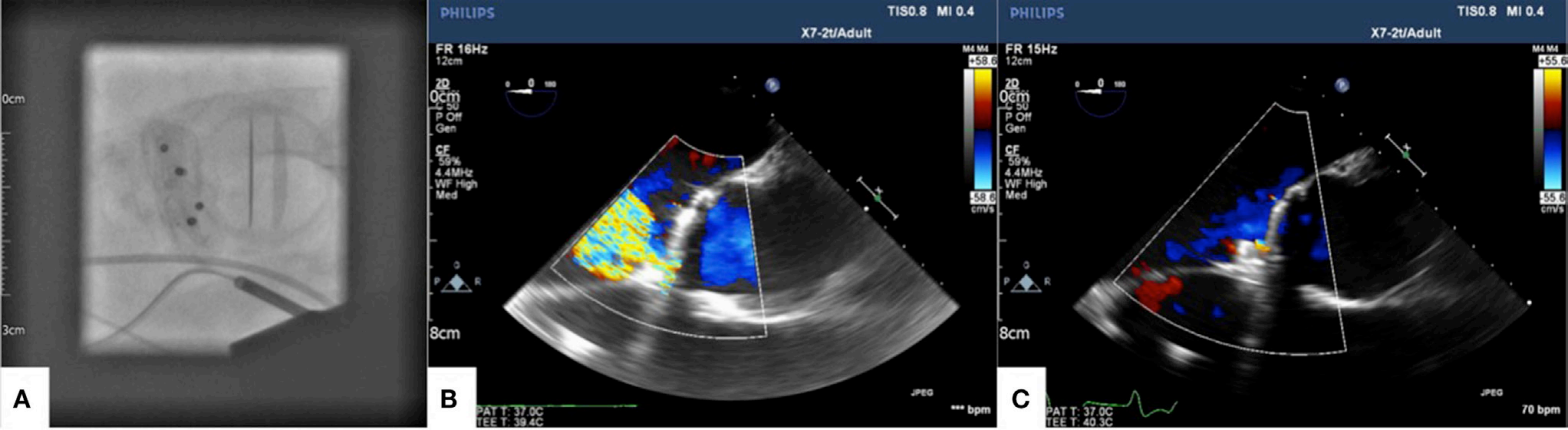
**AVP3 14/5 devices shown in final position in short axis on fluoroscopy (A)**. Color Doppler showing baseline paravalvular leak **(B)** and final result after device closure **(C)** on intraoperative transoesophageal echocardiography.

A left anterolateral minithoracotomy was performed, and the pericardium was opened in the apical region. Two purse string sutures were placed on the left ventricular apex. An 11 F sheath was inserted within the purse strings, and the PVL was crossed with a wire; two AVP3 14/5 mm Amplatzer Vascular Plug devices (St. Jude Medical Inc., St. Paul, MN, USA) were then implanted to close the leak (Figure 3A). Intraoperative transesophageal echocardiogram demonstrated a good final result with residual trivial paravalvular regurgitation (Figures 3B,C show baseline PVL and final result, respectively).

The patient was discharged home 7 days after the procedure. Laboratory findings still suggested some degree of hemolytic anemia, with increased indirect bilirubin, increased LDH, and reduced haptoglobin. Nevertheless, in respect to his heart failure, there had been symptomatic improvement at follow-up. Transthoracic echocardiography, performed 4 days and 2 months postoperatively, demonstrated severely reduced left ventricular function with no paravalvular regurgitation.

## Discussion

Adult patients with CHD represent a challenging population due to increased technical complexity and high-risk comorbidities. Therefore, hybrid procedures are becoming increasingly popular in the treatment of a wide range of conditions in this cluster of patients.

1. Transcatheter pulmonary valve implantation has emerged as an alternative to surgical pulmonary valve replacement. Currently, two devices are available for TPVI: the Melody valve (Medtronic, Minneapolis, MN, USA) and the Edwards Sapien XT valve (Edwards Lifesciences, Irvine, CA, USA) (4). The Melody valve has been validated for RVOTs up to 24 mm in diameter (5, 6), while the Edwards Sapien XT valve is available in 23, 26, and 29 mm sizes. The first patient presented in this paper had a dilated RVOT, with a diameter of 47 mm. Because of multiple comorbidities, it was decided not to replace the pulmonary valve with conventional surgery, which would have required CPB. However, the native RVOT was not suitable for a percutaneous TPVI due to an excessively dilated main pulmonary artery (47 mm). For this reason, plication of the main pulmonary artery was required. Furthermore, plication is not sufficient to create an appropriate landing zone for the Edwards Sapien XT valve; as a consequence, it is common practice to stent the native the RVOT before valve implantation (7). Therefore, we gained access for the main pulmonary artery plication *via* redo median sternotomy, which also allowed implantation of the RVOT stent and the Edwards Sapien XT prosthesis *via* a perventricular approach. At our institution, we have gained significant experience in hybrid pulmonary valve replacement, primarily in the context of pulmonary regurgitation after tetralogy of Fallot repair (8). In a retrospective study published in 2013, we compared the results of hybrid off-pump perventricular pulmonary valve implantation with conventional pulmonary valve replacement: significant differences were found in favor of the hybrid approach with regards to operative time, postoperative hemoglobin level, postoperative bleeding, and need for blood products (9).
2. Partial anomalous pulmonary venous drainage is an uncommon congenital anomaly, with incidence reported to be between 0.4 and 0.7% in autopsy series. PAPVD with intact septum accounts for about 25–35% of all PAPVD cases (10, 11). Furthermore, surgical treatment is safe, and both early and late events, including venous pathway stenosis, are rare (12). Hence, there is no widely agreed interventional management for baffle obstruction. A conventional surgical approach to address this condition requires not only CPB but also aortic cross-clamp and cardioplegic arrest. Our second patient had undergone a PAPVD repair with transection and relocation technique; as a consequence, the stump of the superior vena cava had been converted into the inflow of the pulmonary veins into the left atrium at the time of the first operation. Approaching this anterior structure through a redo median sternotomy necessitated minimal dissection and provided direct access to the pericardial baffle for the interventional cardiologist.
3. The incidence of paravalvular regurgitation after aortic and mitral valve replacement surgery is reported to be 2–17% (13, 14). About 3% of all patients with paravalvular regurgitation require an interventional treatment because of heart failure, hemolysis, or both (14). Although reoperative surgery is currently the gold standard treatment for PVL, it carries high early and mid-term morbidity and mortality and significant risk of recurrence (15, 16). Various percutaneous and hybrid techniques have been developed over the last years to address paravalvular regurgitation avoiding redo sternotomy, CPB, and aortic cross-clamp (14, 17). Randomized controlled trials comparing surgical and catheter closure of PVL are not currently available; however, early mortality in elective cases was reported to be 2.9% in a recent large series from UK and Irish centers (18). To our knowledge, there are no published series reporting results of transcatheter PVL closure in adults with previous diagnosis of atrioventricular septal defect. Although technical aspects of transapical PVL closure in such patients are probably the same that apply for patients who underwent mitral valve replacement for acquired valve disease, long-term results may be different due to different annular structure in the CHD group. In this paper, we presented a patient with both heart failure and hemolysis and severe PVL on a left atrioventricular valve mechanical prosthesis. We chose to close the PVL through a transcatheter transapical approach, in order to avoid a third redo sternotomy in a patient with significant left ventricular dysfunction. The procedure was uneventful, and the patient was discharged from the hospital with no obvious left atrioventricular valve regurgitation.

## Concluding Remarks

The choice of the optimal treatment strategy in adult patients with CHD often represents a challenge for cardiac surgeons and cardiologists. This is a heterogeneous population; hence, precise guidelines are seldom available to advise clinicians.

According to our experience, hybrid surgery is a promising therapeutic modality and it broadens the spectrum of therapeutic options for this group of patients characterized by a combination of complex anatomy and high preoperative risk. A multidisciplinary and patient-tailored approach is crucial in these complex clinical scenarios.

## Informed Consent

Patients involved in this manuscript have signed written informed consent to collect and store their clinical details in our database for clinical and scientific purposes.

## Author Contributions

Substantial contributions to the conception or design of the work: FR, DK, MT, AP, SS, OU, and MC. Drafting the work or revising it critically for important intellectual content: FR, DK, MT, AP, SS, OU, and MC. Final approval of the version to be published: FR, DK, MT, AP, SS, OU, and MC. Agreement to be accountable for all aspects of the work in ensuring that questions related to the accuracy or integrity of any part of the work are appropriately investigated and resolved: FR, DK, MT, AP, SS, OU, and MC.

## Disclaimer

This article/paper/report presents independent research funded by the National Institute for Health Research (NIHR). The views expressed are those of the author(s) and not necessarily those of the NHS, the NIHR, or the Department of Health.

## Conflict of Interest Statement

The authors declare that the research was conducted in the absence of any commercial or financial relationships that could be construed as a potential conflict of interest.
